# Single-molecule real-time sequencing facilitates the analysis of transcripts and splice isoforms of anthers in Chinese cabbage (*Brassica rapa* L. ssp. *pekinensis*)

**DOI:** 10.1186/s12870-019-2133-z

**Published:** 2019-11-27

**Authors:** Chong Tan, Hongxin Liu, Jie Ren, Xueling Ye, Hui Feng, Zhiyong Liu

**Affiliations:** College of Horticulture, Shenyang Agricultural University, Shenyang, Liaoning 110866 People’s Republic of China

**Keywords:** Chinese cabbage, Anther, Full-length transcript, Alternative splicing, Fusion transcript

## Abstract

**Background:**

Anther development has been extensively studied at the transcriptional level, but a systematic analysis of full-length transcripts on a genome-wide scale has not yet been published. Here, the Pacific Biosciences (PacBio) Sequel platform and next-generation sequencing (NGS) technology were combined to generate full-length sequences and completed structures of transcripts in anthers of Chinese cabbage.

**Results:**

Using single-molecule real-time sequencing (SMRT), a total of 1,098,119 circular consensus sequences (CCSs) were generated with a mean length of 2664 bp. More than 75% of the CCSs were considered full-length non-chimeric (FLNC) reads. After error correction, 725,731 high-quality FLNC reads were estimated to carry 51,501 isoforms from 19,503 loci, consisting of 38,992 novel isoforms from known genes and 3691 novel isoforms from novel genes. Of the novel isoforms, we identified 407 long non-coding RNAs (lncRNAs) and 37,549 open reading frames (ORFs). Furthermore, a total of 453,270 alternative splicing (AS) events were identified and the majority of AS models in anther were determined to be approximate exon skipping (XSKIP) events. Of the key genes regulated during anther development, AS events were mainly identified in the genes *SERK1*, *CALS5*, *NEF1*, and *CESA1/3*. Additionally, we identified 104 fusion transcripts and 5806 genes that had alternative polyadenylation (APA).

**Conclusions:**

Our work demonstrated the transcriptome diversity and complexity of anther development in Chinese cabbage. The findings provide a basis for further genome annotation and transcriptome research in Chinese cabbage.

## Background

Gene sequencing emerged as a revolutionary technology in the field of biological research. The first of these technologies was Sanger sequencing; however, due to low throughput and poor automation, Sanger sequencing was severely limited in its application in genome and transcriptome analysis [1]. The advent of NGS technologies, such as ABI SOLiD, Illumina Solexa, and Roche 454 systems, stimulated structural and functional genomics studies for diverse plant species. Among these technologies, Illumina sequencing has the advantages of high accuracy, high throughput, high sensitivity, and low cost, and is now the most widely used platform in genome sequencing [2]. *C. sativus* was the first vegetable crop to complete genome-wide de novo sequencing by NGS. Subsequently, the main crop genomes of *S. tuberosum*, *T. aestivum*, *B. napus*, *G. raimondii*, and other crops were sequenced. Short-read RNA-Seq by NGS is frequently applied for transcriptome analysis. Using short-read RNA-Seq, researchers can obtain profiles for genome-wide expressed genes, including low-abundance genes, as well as new genes and SNPs [3]. Research on gene expression profiling of pollen and anther development in the genus *Brassica* has accumulated in recent years [4–11]. However, although NGS technologies are effective, they still have several drawbacks, including the generation of relatively short reads, which may lead to misassembly and gaps [12]. Moreover, short reads are not well suited to accurately detecting structural variations (SVs) and transcript isoforms generated by AS events [13, 14]. Limited by NGS methods, short RNA-Seq reads must be assembled into longer DNA contigs [15], a process that is susceptible to misassembly of short sequence reads transcribed from highly repetitive regions or similar members of multiple gene families [16]. This problem may become even more severe for polyploid plants that often harbor higher sequence similarity between coexisting subgenomes, which frequently indirectly leads to annotation error. Moreover, short-read RNA-Seq cannot distinguish between alternatively spliced forms for individual transcripts, which can make up a large proportion of transcripts. For instance, approximately 83.4% of multiple-exon genes are subject to AS in *A. thaliana*, which contributes to organismal protein diversity without massively increasing the number of genes [17].

Third generation sequencing (TGS) technologies have recently been developed, which is known for single-molecule sequencing (SGS) and sequencing in real-time [18]. The first TGS technology platform was delivered by Helicos Biosciences, but it proved unworkable from the market because it was relatively slow, expensive, and generated short reads (~ 32 bp) [19]. Soon after, single-molecule real-time sequencing (SMRT) sequencing by PacBio emerged as unique opportunity for constructing full-length transcripts [20]. The distinguishing features of SMRT technology is the production of long reads. Initially, the average length of reads generated by SMRT technology was just ~ 1.5 kb, but is now 10-15 kb [21]. Therefore, SMRT can improve the accuracy of gene models as it allows generation of reads that cover full-length transcripts [14]. However, SMRT sequencing still has major technical defects and limitations, namely its relatively high cost, lower throughput, and high error rate. Therefore, at present, a combination of NGS technologies and SMRT sequencing is preferable: consensus sequence reads are constructed from raw PacBio subreads and aligned with the reads generated from appropriate NGS platforms. Using this approach, multiple complex genomes have been successfully de novo assembled or improved [22–30].

SMRT sequencing has been previously effectively applied to transcriptome analysis. Well-characterized full-length transcripts are not only beneficial for analysis of gene structure and alternative splicing, but also greatly improve functional studies of important loci [15]. Early applications of SMRT sequencing on the transcriptome were relatively narrow, and most focused on model organisms such as humans [13] and yeast [31]. Since 2015, SMRT technology has been widely applied to characterize the full-length sequence of genome and transcripts in diverse species. SMRT has facilitated structural genomics and grain transcriptome research in common hexaploid wheat [15]. In danshen, the application of SMRT sequencing to different root tissues revealed that about 40% of the detected gene loci had occurred alternative splicing (AS) events [32]. In maize B73, over 111,000 transcripts from six tissues were identified, unveiling the complexity of the transcriptome by SMRT sequencing [14]. PacBio SMRT was employed for the sorghum transcriptome, and over 11,000 novel splice isoforms, alternative polyadenylation (APA) of ~ 11,000 expressed genes, and more than 2100 novel genes were uncovered at an unprecedented scale [33]. The *A. thaliana* transcriptome was analyzed by SMRT, enhancing the understanding of differentially expressed AS isoforms under normal conditions and in response to ABA treatment [17]. In moso bamboo, over 42,280 distinct splicing isoforms and 25,069 polyadenylation sites were found [34]. In *Dendrobium officinale*, the full-length cDNA transcripts of stems and leaves uncovered multiple genes involved in polysaccharide synthesis [35]. The red clover transcriptome was analyzed by SMRT sequencing and the results uncovered about 29,730 novel isoforms from known genes and 2194 novel isoforms from novel genes, in addition to over 5000 AS events, over 4300 long non-coding RNAs (lncRNAs), and 3700 fusion transcripts [36]. Using SMRT technology, a total of 113,321 transcripts were obtained from alfalfa leaves from three different development stages; sequencing data uncovered about 7568 AS events and 17,740 lncRNAs [37]. The above works are crucial for providing deeply understanding of their genome and transcripts.

The genus *Brassica* comprises multiple economically important vegetable and oil crops*.* The ‘triangle of U’ is well established and refers to three diploid species, *B. rapa* (A genome, 2n = 20), *B. nigra* (B genome, 2n = 16), and *B. oleracea* (C genome, 2n = 18), as well as three amphidiploid species, *B. napus* (AC genome, 2n = 38), *B. juncea* (AB genome, 2n = 36), and *B. carinata* (BC genome, 2n = 34). In 2011, the first genus *Brassica* genome draft, *B. rapa* genome v1.5, was published. The 283.8 Mb genome was generated using next-generation sequencing (NGS) technology with a contig N50 size of 46 kb, and greatly facilitated genomics and molecular biology research, as well as the generic breeding of *B. rapa* and other *Brassica* species [38]. The second version (v2.0) was assembled in 2017. Further improving the scaffold order, the upgraded *B. rapa* genome v2.5 was 389.2 Mb with a contig N50 size of 53 kb [39]. However, restricted by the read length of NGS technology, the above genome versions had the disadvantages of poor continuity, assembly errors, and low assembly rate of repetitive sequences. A more recent release, *B. rapa* genome v3.0 was de novo assembled and re-annotated using single-molecule sequencing (PacBio), optical mapping (BioNano), and chromosome conformation capture (Hi-C) technologies. The total length of *B. rapa* genome v3.0 was 353.14 Mb, with a contig N50 size of 1.45 Mb and a scaffold N50 size of 4.45 Mb, including 1301 scaffolds and 389 gaps [40]. The high-quality reference genomic information lays a solid foundation for the development of genetics and functional genomics of *B. rapa*, especially the cloning of important agronomic trait regulatory genes and the analysis of genetic background. Only by analyzing the molecular mechanism of trait formation at the genetic level, can we carried out targeted genetic breeding, molecular marker-assisted breeding and even molecular design breeding, greatly improve breeding efficiency and accelerate the cultivation of excellent new varieties.

Owing to broad adaptability and numerous end-uses, Chinese cabbage is the most widely cultivated and consumed vegetable within *B. rapa*. Although the reference genome has been improved using the PacBio Sequel platform, sequence and structural data of tissue-specific mRNA remains scarce in Chinese cabbage. The main objective of this study was to characterize full-length transcripts in Chinese cabbage anthers using the emerging SMRT sequencing technology to unveil the transcriptome complexity of anther development. SMRT sequencing data, corrected by short-read NGS technology, were used to analyze full-length transcripts in anthers to further reveal AS events, lncRNAs, and fusion isoforms in Chinese cabbage. This study provides a valuable resource for further genome re-annotation and increases our understanding of the anther transcriptome.

## Results

### Transcriptome sequencing and error correction

**Fig. 1:**
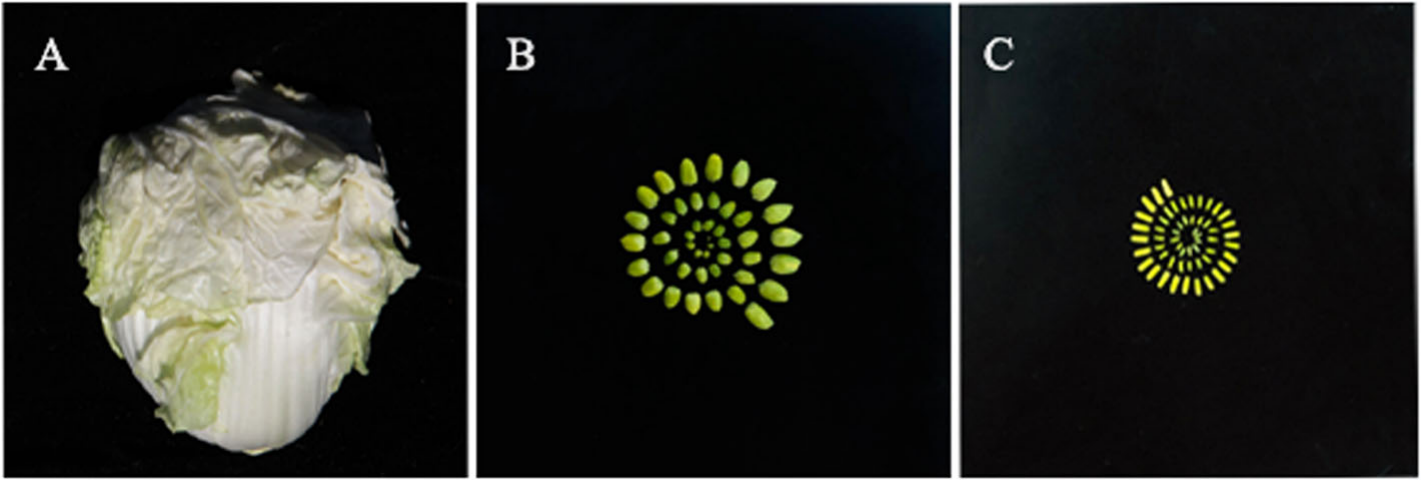
Morphological characteristics of DH line ‘FT’. **a** Leafy head. **b** The entire buds of inflorescence. **c** Anthers during different development stages

**Fig. 2:**
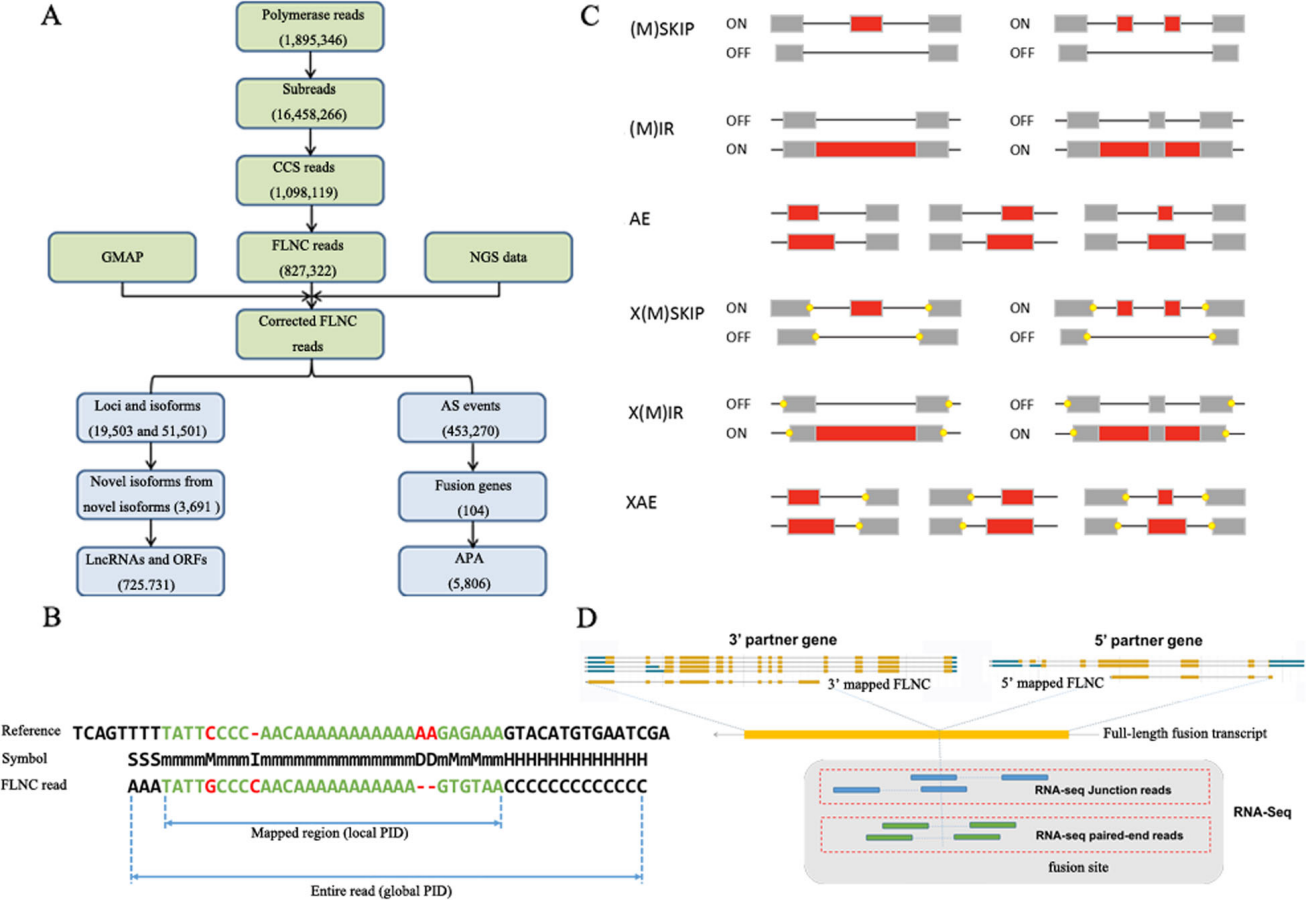
Bioinformatics analysis. **a** Flow chart of PacBio Sequel platform. **b** Illustration of FLNC mapping and PID calculation. m: match. M: mismatch; S: soft-clipping. H: hard-clipping. D: deletion; I: insertion. The calculation formulas of PID were as follow: local PID = m/ (m + M + D + I), gobal PID = m/ (m + M + S + H + D + I). **c** Classification map of AS events. (M)SKIP: (cassette exons) exon skipping; (M)IR: retention of (multiple) single introns; AE: alternative exon ends (5′, 3′ or both); X(M)SKIP: approximate (cassette exons) exon skipping; X(M)IR: approximate retention of (multiple) single introns; XAE: approximate alternative exon ends; **d** Schematic diagram of fusion transcripts detection

Limited by the capacity of short-read RNA-Seq on an Illumina platform, anther-specific transcriptome analysis of Chinese cabbage double haploid (DH) line ‘FT’ (Fig. 1 a-c) was carried out using the PacBio Sequel platform. To identify the transcripts as completely as possible, high-quality total mRNAs from each of the pooled samples obtained throughout anther development were extracted and mixed to obtain full-length sequences and splice variants. The entire flow is shown in Fig. 2a.

**Fig. 3:**
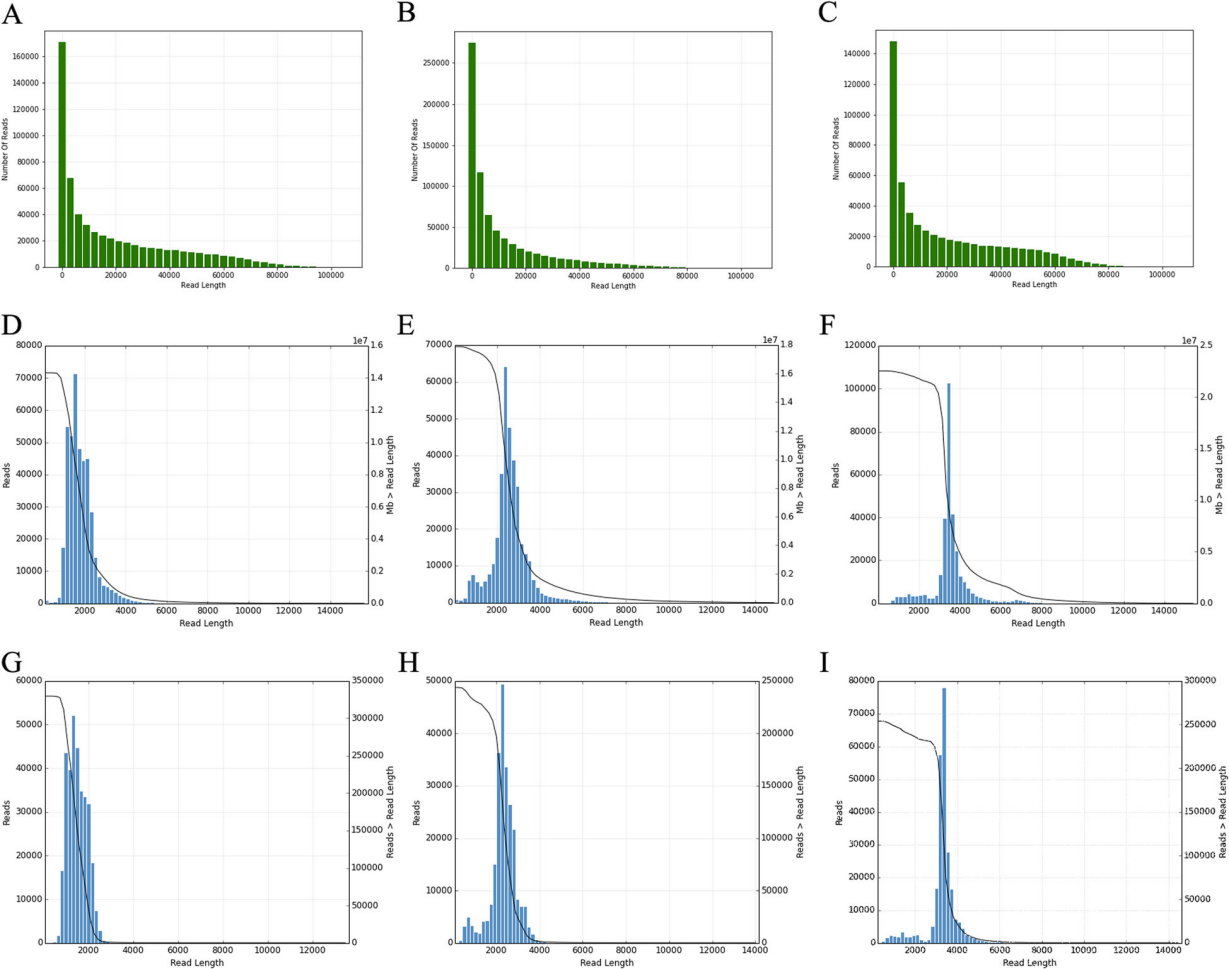
Length distribution of the PacBio Sequel data output. **a-c** Number and length distribution of polymerase reads. **d-f** Number and length distribution of CCSs. **g-i** Number and length distribution of FLNC reads

Three different SMRT bell libraries were constructed and sequenced using the PacBio Sequel platform with cDNA insert sizes 1–2 kb, 2–3 kb, and > 3 kb. After filtering, a total of 1,895,346 polymerase reads representing more than 33.14 G bases were captured, with a mean length of 17,965 bp and N50 of 39,750 bp (Additional file 2: Table S1; Fig. 3a-c). After removing the adapter from polymerase reads, approximately 16,458,266 filtered subreads were obtained with a mean length of 2121 bp (Additional file 2: Table S2). A total of 1,098,119 circular consensus sequences (CCSs) with an average depth of 11.33 passes in three libraries were generated from subreads after merging and error correction by multiple sequencing (Additional file 2: Table S3). The length distribution of CCSs was consistent with the expected size of the three libraries (Fig. 3d-f). CCSs were counted separately as follows: 5′ primer, 3′ primer, poly-A tail, full-length, and full-length non-chimeric (FLNC). In total, we detected 863,281 full-length reads, containing the 5′ primer, 3′ primer, and poly-A tail. Then, 827,322 reads were considered to be FLNC with low artificial concatemers, accounting for 75.34% of CCSs (Table 1). The mean length of FLNC reads in the 1-2 K, 2-3 K, and > 3 K libraries were 1499 bp, 2324 bp, and 3288 bp, respectively (Fig. 3g-i; Table 1). The quantity distribution of FLNC reads in each library was similar, although the 1–2 kb library was slightly larger than the other two libraries. Overall, we have comprehensively obtained full-length transcripts, making it possible to accurately construct splice variants.

**Table 1:** Summary of ROI from the PacBio Sequel platform

Sample	Library	Cell	CCS	5′ primer	3′ primer	Poly-A	Full-length	FLNC	Mean FLNC length (bp)
Ant 1-2 k	1-2 K	D01	418,042	390,507	386,739	365,528.00	352,741	329,694	1499
Ant 2-3 K	2-3 K	E01	355,765	296,959	290,222	270,230.00	246,734	243,747	2324
Ant > 3 K	3 K+	A01	324,312	298,840	295,242	279,866.00	263,806	253,881	3288
Total	–	–	1,098,119	986,306	972,203	915,624.00	863,281	827,322	–

**Fig. 4:**
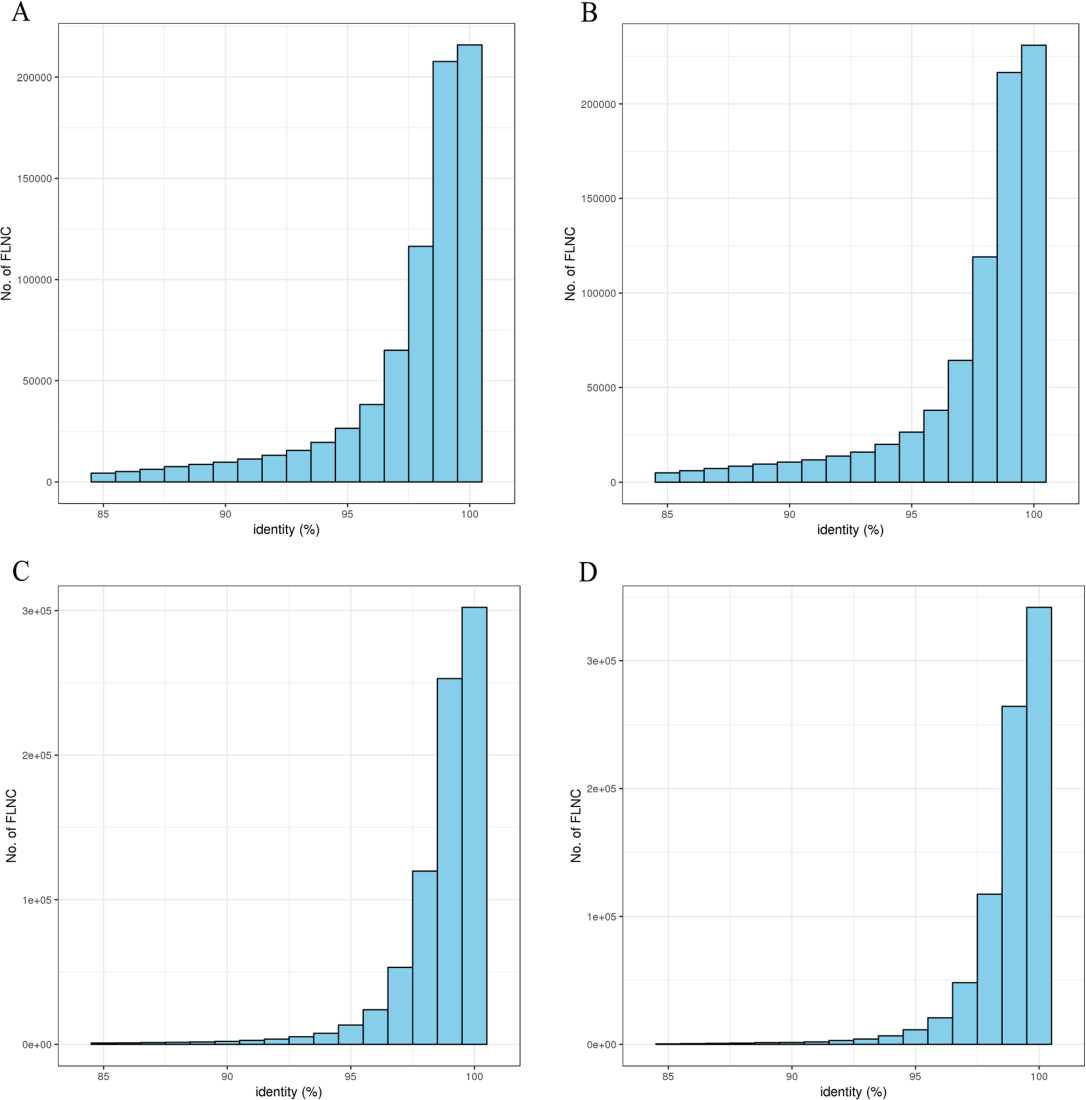
The distribution of PID (percentage-of-identity) before and after error correction. **a** Gobal PID distribution before error correction. **b** Local PID distribution before error correction. **c** Gobal PID distribution after error correction. **d** Local PID distribution after error correction

SMRT sequences have a high base error rate—up to 12–15%—mainly due to the extra insertion of bases. To further correct the FLNC reads sequenced by the PacBio Sequel platform, Illumina HiSeq 2000 transcripts of the anthers were employed by the proovread software. Using GMAP^2^ software, the FLNC reads before and after error correction were compared to the reference genome for counting global and local percentage-of-identity (PID) (Fig. 4). Before error correction, the mean global PID was 94.97%. After error correction, the value was up to 97.04% (Additional file 2: Table S4). After updating, we obtained 725,731 high-quality FLNC reads for subsequent investigation (Table 2).

**Table 2:** Classification of reference genome comparison results

Type	Pre-correction	Post-corrrction	Merge
Unmapped	3146 (0.38%)	616 (0.07%)	558 (0.07%)
Multiple-best map	7212 (0.87%)	7550 (0.91%)	6782 (0.82%)
Low PID map	230,229 (27.83%)	99,570 (12.04%)	94,251 (11.39%)
High quality map	586,735 (70.92%)	719,586 (86.98%)	725,731 (87.72%)

### Loci and isoform detection and characterization

**Fig. 5:**
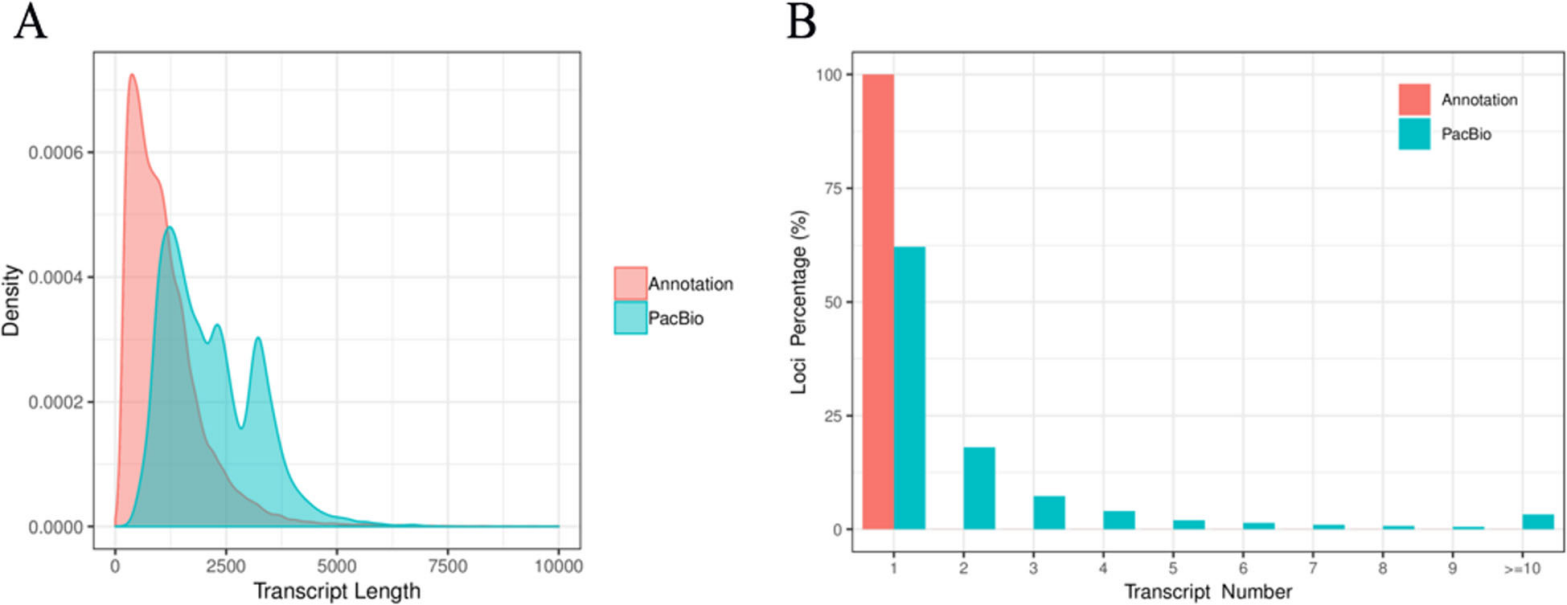
Isoform length density and Isoform number of loci density. **a** The length distribution of all isoforms in the PacBio Sequel platform compared to the reference genome. **b** The number distribution of isoforms from each locus in the PacBio Sequel platform compared to the reference genome

Error correction analysis allows accurate mapping of FLNC reads to the reference genome, including start site, termination site, and splicing site. Based on this information, gene loci and isoform can be identified. To assess isoform length density, we compared the loci coverage of the PacBio data set with the *B. rapa* v3.0 annotation. In our data, a total of 725,731 error-corrected FLNC reads covered 51,501 isoforms and were allocated to 19,503 loci. About 9102 loci were 1–2 kb in length, followed by 2–3 kb (3867), > 3 kb (3355), and < 1 kb (3179). In the reference genome, about 46,250 isoforms covered 46,250 loci, and the loci were mostly distributed at < 1 kb (24,937), followed by 1–2 kb (15,700), 2–3 kb (3959), and > 3 kb (1654) (Table 3; Fig. 5a). Similarly, we evaluated the isoform number of loci density, indicating that each locus could produce a unique isoform in the reference genome. However, in our data, approximately 12,124 (62.16%) loci could produce a unique isoform and more than five isoforms covered about 6.83% of the PacBio annotation loci (Fig. 5b). The gene A06.1469 had the largest number of isoforms at ~ 524. Thus, the PacBio data set could provide richer isoform length diversity and isoform number of loci density than the reference genome, which helped more fully reveal the complexity of the anther transcriptome. In addition, we evaluated the exon-intron structure of each loci and isoform obtained by PacBio Sequel platform. Among the 19,503 loci, there were 2911 (14.93%) single-exon loci and 16,592 (85.07%) multiple exon loci. Out of 51,501 isoforms, 4.188 (8.13%) were single exon, and 47,313 (91.87%) were multiple exon (Table 3).

**Table 3:** Gene structure annotation

Type	Loci < 1 K	Loci 1-2 K	Loci 2-3 K loci	Loci > = 3 K	Total Loci	Single-exon loci	Multiple-exon loci	Total isoform	Single-exon isoform	Mutiple-exon isoform
Reference annotation	24,937 (53.92%)	15,700 (33.95%)	3959 (8.56%)	1654 (3.58%)	46,250	11,102 (24.00%)	35,148 (76.00%)	46,250	11,102 (24.00%)	35,148 (76.00%)
PacBio data set	3179 (16.30%)	9102 (46.67%)	3867 (19.83%)	3355 (17.20%)	19,503	2911 (14.93%)	16,592 (85.07%)	51,501	4188 (8.13%)	47,313 (91.87%)

Based on the characteristics of library construction, we could not guarantee the structural integrity of the 5′ end of the transcripts. Therefore, the full-length evaluation of FLNC and isoforms produced by PacBio Sequel platform were only estimated at the 5′ end. With multiple-exon transcripts of genome annotation as a reference, isoforms obtained from the PacBio data set with identical direction and overlap greater than 20% were screened. If the first splice donor site at 5′ end of genome annotation was indeed included at the first splice donor site of the isoform obtained from PacBio data set, then the isoform was considered to be a full-length isoform, and the corresponding FLNC was considered to be a full-length FLNC. Our data indicated that approximately 76.66% multiple-exon isoforms and 88.22% multiple-exon FLNC contained the same splice donor site at the 5′ end as the reference annotation, and were regarded as full length, implying a relatively high integrity in structure (Additional file 2: Table S5).

**Fig. 6:**
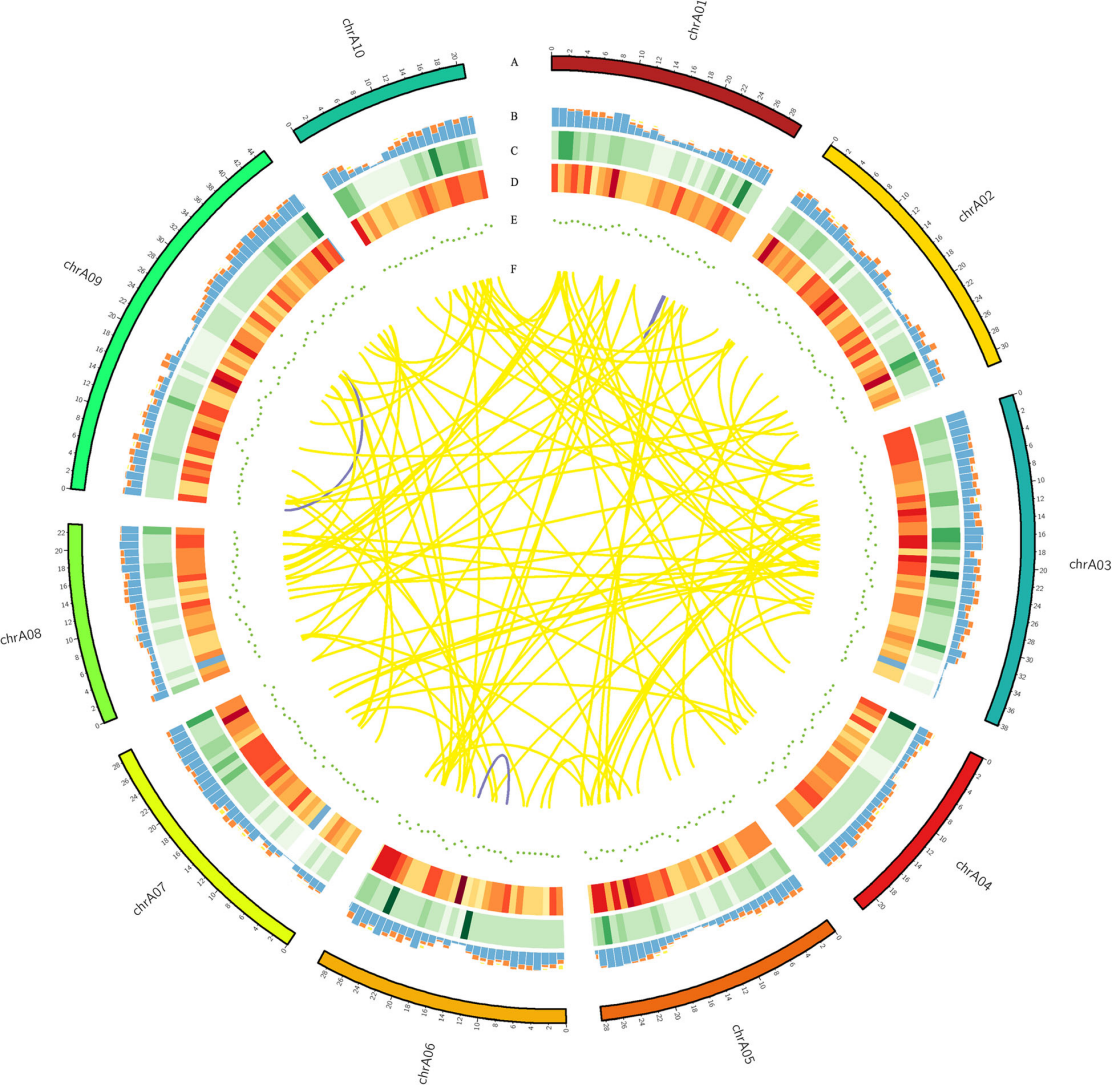
Circos visualization of the PacBio Sequel platform at genome-wide level. **a** Ten chromosomes distribution of *B.rapa* genome. **b** APA sites distribution mapped to *B.rapa* genome. **c** Novel isoforms density from the PacBio Sequel platform. **d** Novel loci density from the PacBio Sequel platform. The closer the color is to red, the higher the density. Conversely, the closer the color is to blue, the lower the density. **e** LncRNA density from the PacBio Sequel platform. The closer the point is to the center, the lower the density. **f** Fusion transcripts distribution. Purple line represents intra-chromosome fusion transcripts, and yellow line represents inter-chromosomal

Next, the sequenced gene loci and isoforms were compared with the reference annotation to determine novel loci or novel isoforms. The published *B. rapa* genome annotation contains 46,250 loci with 46,250 isoforms. In our PacBio data set, out of 51,501 isoforms from 19,503 genes, we identified 16,821 known isoforms from known genes. In addition, 2682 transcripts overlapping with no annotated gene were considered likely to be novel genes (Additional file 2: Table S6; Fig. 6d). Those novel genes were found to generate 3691 novel isoforms (Fig. 6c). We also found 38,992 novel isoforms from 11,398 known genes. Of the 3691 novel isoforms, 1455 (39.42%) were single-exon isoforms and 2236 (60.58%) were multiple-exon isoforms. The above novel genes and isoforms were beneficial to the improvement of the integrity of the *B. rapa* genome annotation.

### Functional annotation of novel isoforms

**Fig. 7:**
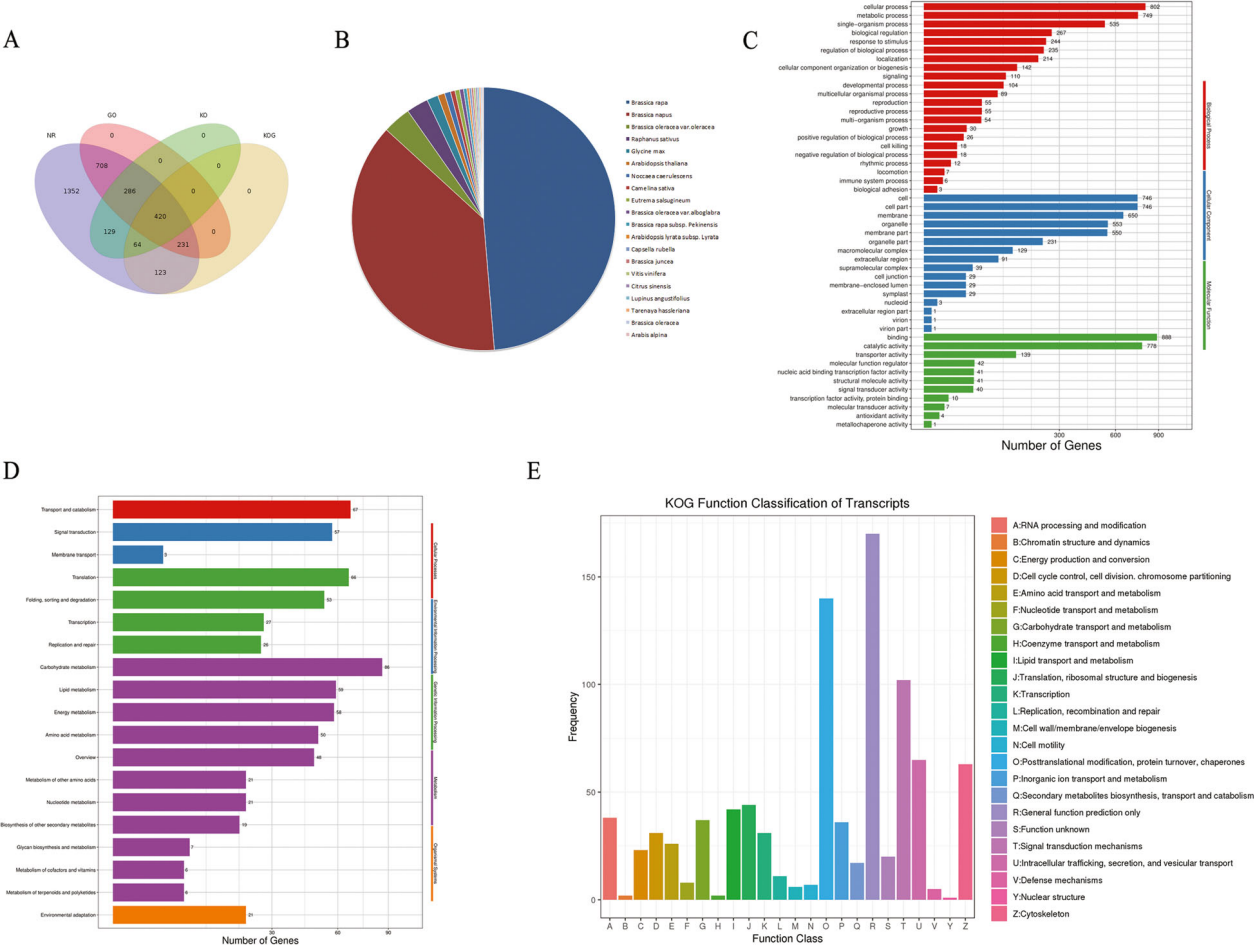
Function annotations of novel isoforms identified by the PacBio Sequel platform. **a** The number statistics of novel isoforms in Nr, GO, KEGG, KOG databases. **b** Distribution of novel isoforms in Nr homologous top 20 species. **c** Distribution of novel isoforms in GO terms. **d** Distribution of novel isoforms in KEGG pathway. **e** Distribution of novel isoforms in KOG

In this study, all 3691 novel isoforms were functionally annotated by searching NCBI non-redundant protein sequences (NR) (89.76%), Gene Ontology (GO) (44.57%), EuKaryotic Orthologous Groups (KO) (24.36%), euKaryotic Ortholog Group (KOG) (22.70%), and Swiss-Prot Protein Sequence (Swiss-Prot) databases (42.81%), and a total of 377 (10.21%) were unannotated (Additional file 2: Table S7). A total of 420 novel isoforms had significant hits in all five databases (Fig. 7a). In the NR database, the largest three groups of novel isoforms were distributed in *B. rapa* (1578), *B. napus* (1236), and *B. oleracea* (112) (Fig. 7b). GO analysis assigned the enrichment of 1645 isoforms to three ontologies, namely, biological process, cellular component, and molecular function. We found 1602 GO terms in “biological process,” of which “cellular process” (48.75%), “metabolic process” (45.53%), and “single-organism process” (32.52%) accounted for a large proportion. Many of the terms in “biological process” were associated with anther development, such as pollen germination, fatty acid metabolic process, pollen exine formation, pollen tube development, anther dehiscence, pollination, and pollen-postil interaction. A total of 358 GO terms were detected in “cellular component,” with “cell” (45.40%), “cell part” (45.40%), and “membrane” (39.51%) the largest three enrichment terms. Eleven and two novel isoforms could be assigned to the GO terms “pollen tube tip” and “pollen tube,” respectively in “cellular component.” Our data showed that 769 GO terms were assigned to “molecular function”, and the most highly abundant terms were “binding” (53.98%) and “catalytic activity” (47.29%) (Fig. 7c). To identify the enrichment pathways, a total 899 novel isoforms were subjected to 101 KEGG pathways. Novel isoforms in KEGG pathways consisted of five hierarchy: “cellular processes”, “environmental information processing”, “genetic information processing”, “metabolism and “organismal systems”. Among these terms, the most abundant hierarchy was “metabolism” (71.29%), followed by “genetic information processing” (11.88%) (Fig. 7d). In addition, we found three metabolic pathways associated with “fatty acid”, which were essential for anther development [41]. KOG analysis shown that 838 novel isoforms were assigned to 24 categories, and the largest three classes were “general functional prediction only” (20.29%), “posttranslational modification, protein turnover, chaperones” (16.71%), and “signal transduction mechanisms” (12.18%) (Fig. 7e).

### LncRNA and ORF prediction of novel isoforms

**Fig. 8:**
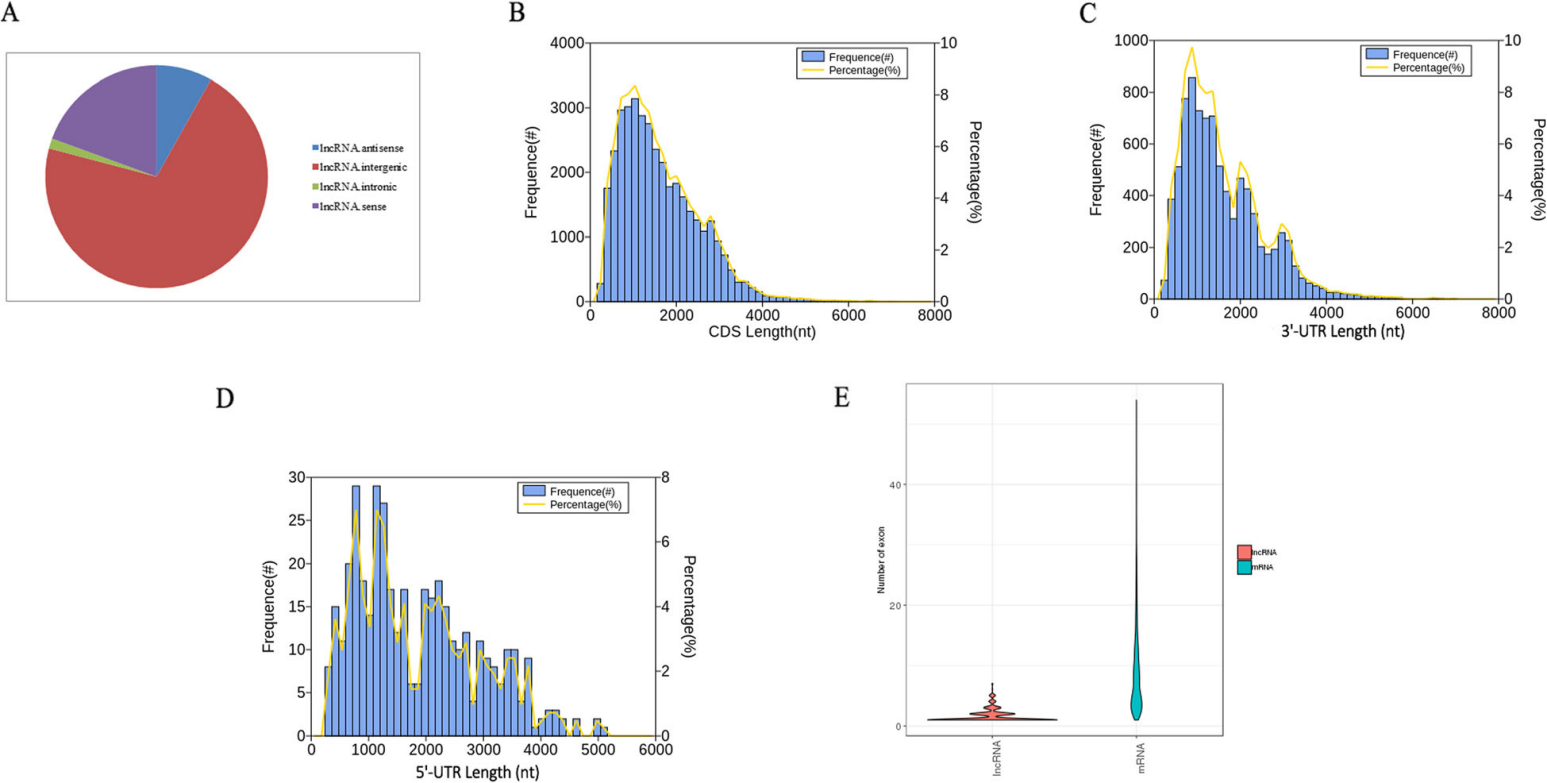
LncRNA and ORF analysis. **a** Identification of four types of lncRNA. **b** Number, percentage and length distributions of CDS of novel isoforms with predicted ORF. **c** Number, percentage and length distributions of 3′ UTRs of novel isoforms with predicted ORF. **d** Number, percentage and length distributions of 5′ UTRs of novel isoforms with predicted ORF. **e** Exon number distribution of novel isoforms with predicted ORF and lncRNAs

LncRNAs have regulatory functions, and are crucial for post-transcription, transcription, and epigenetics [42]. The novel isoforms from novel genes and novel isoforms from known genes with no hit in the above functional annotation databases, were predicted by CPAT software to identify lncRNAs in the PacBio data set. To obtain a high confidence set of lncRNAs, we retained the isoforms with an optimum cutoff value, and that were more than 200 bp in length. A total of 407 novel isoforms were predicted to be lncRNAs, accounting for 1% of all novel isoforms, with a mean length of 1127 bp (Additional file 2: Table S8). About 168 lncRNAs (41.28%) were longer than 1000 bp, and four lncRNAs were longer than 4000 bp. The predicted lncRNAs were classified into four types, consisting of 33 antisense (8.11%), 289 intergenic (71.01%), 6 intronic (1.47%), and 79 sense (19.41%) lncRNAs (Fig. 8a). Mapping of the predicted lncRNAs to *B. rapa’s* ten chromosomes was presented using Circos visualization software, revealing that 407 lncRNAs were randomly distributed, of which three lncRNAs were not anchored on the chromosomes (Fig.6e). The open reading frames (ORFs) were predicted by transDecoder software, resulting in 37,549 novel isoforms with a predicted ORF. Next, the density and length distributions of coding sequences (CDS) were investigated, and the mean length was 915 bp (Fig. 8b). The encoded peptide sequences are listed in Additional file 2: Table S9. The density and length of the distributions of the 5′ and 3′ boundaries of untranslated regions (UTRs) were identified, and the results revealed 415 3′ UTRs with a mean length of 641 bp and 8791 5′ UTRs with a mean length of 788 bp (Fig. 8c, d). Furthermore, the exon structures of novel isoforms with a predicted ORF and lncRNAs were analyzed, and the average number of exons per mRNA and lncRNA was 8.78 and 1.65, respectively (Fig. 8e).

### Various models of AS

**Fig. 9:**
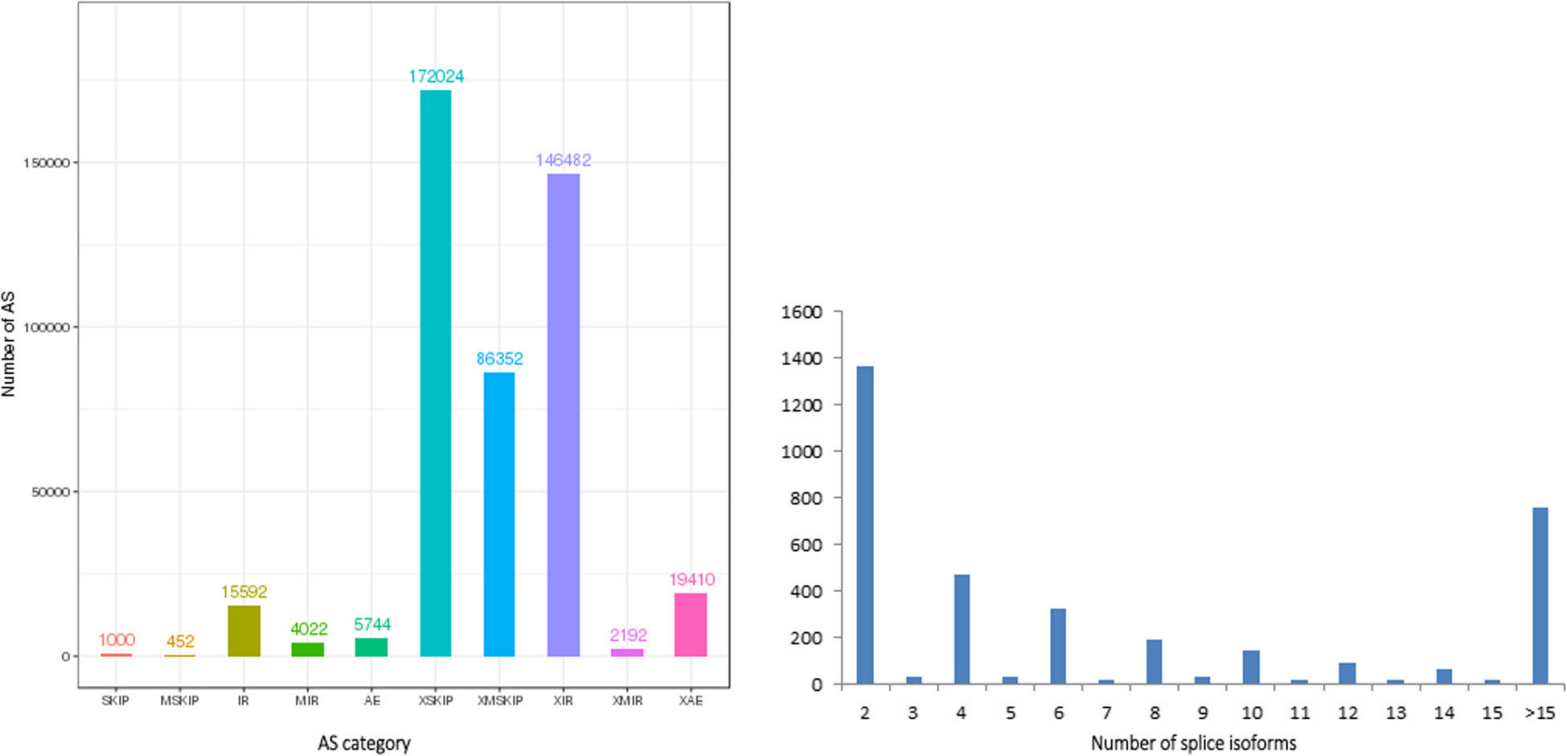
Identification of AS events. **a** The number distribution of AS events in loci detected by the PacBio Sequel platform. **b** Distribution of loci that produce two or more splice isoforms detected by the PacBio Sequel platform

AS increases the diversity of transcriptomes and proteomes according to the different splice modes, rather than by massively amplifying the number of genes in cells or tissues [43, 44]. Traditionally, AS events consisted of several different types: exon skipping (SKIP) and cassette exons (MSIP), retention of single (IR) and multiple (MIR) introns, alternative exon ends (5′, 3′, or both) (AE), approximate exon skipping (XSKIP) and cassette exons (AMSKIP), approximate retention of single (XIR) and multiple (XMIR) introns, and approximate alternative exon ends (XAE) (Fig. 2c). The latest *B. rapa* v3.0 reference genome did not incorporate AS models and splice isoforms. However, there was a total of 156,516 unique splice junctions detected in the earlier *B. rapa* genome v1.5, and IR events were predominant in the reference genome, similar to in species such as *M. truncatula*, *P. trichocarpa*, *A. thaliana*, *O. sativa*, *C. reinhardtii*, and *B. distachyon* [45]. In our study, we compared the PacBio-sequenced isoforms against the *B. rapa* genome v3.0, and found that a total of 19,503 loci corresponding to 51,501 isoforms underwent 453,270 AS events, indicating the distribution of AS events is much high in anthers (Additional file 2: Table S10). The total AS events generated in our study were: 1000 SKIP, 452 MSKIP, 15,592 IR, 4022 MIR, 5744 AE, 172,024 XKIP, 86,352 XMSKIP, 146,482 XIR, 2192 XMIR, and 19,410 XAE (Fig. 9a). Further, we observed that XSKIP (37.95%) was predominant, while MSKIP (0.1%) was the least frequent event. This findings greatly enriched anther transcript information. In our PacBio Sequel platform analysis, two or more isoforms were found in 3576 genes. Ten or more splice isoforms were detected in 1115 genes (Fig. 9b). The largest number of splice isoforms was 64,266, detected in *BraA06g022340.3C*; this gene is a homolog of *Arabidopsis* H(+)-ATPase 8 (AHA8). To verify the accuracy of AS events detected by SMRT, three genes were randomly selected, and gene-specific primers that spanned the predicted splicing events were designed for RT-PCR. The expression results for RT-PCR and Sanger sequencing in anthers were identical to the splice isoforms detected from PacBio data set, which demonstrated that those data were reliable (Additional file 1: Figure S1).

### Fusion transcript and APA identification

A fusion transcript refers to a new gene formed by splicing together two or more separate genes, which were known as chimeric transcripts. The mechanisms leading to the generation of fusion transcripts include genomic structural variation, transposition, or trans-splicing after transcription. In this study, we identified 104 fusion transcripts, involving 187 annotated genes (Additional file 2: Table S11). Fusion transcripts were most frequently distributed on chromosome A03, followed by chromosome A09 and A01. According to the chromosomal distribution, we detected 101 inter-chromosome and 3 intra-chromosome fusion transcripts (Fig. 6f). This result was consistent with those of other species such as maize [14] and red clover [39]. Previous studies have indicated that most fusion transcripts are composed of two genes [46]. Consistent with these studies, all the 104 fusion transcripts in our data were composed of two genes. In addition, three fusion transcripts detected by SMRT were randomly selected and experimentally validated in anther and four other floral organs. The experimental results confirmed the authenticity of these chimeric RNAs (Additional file 1: Figure S1).

**Fig. 10:**
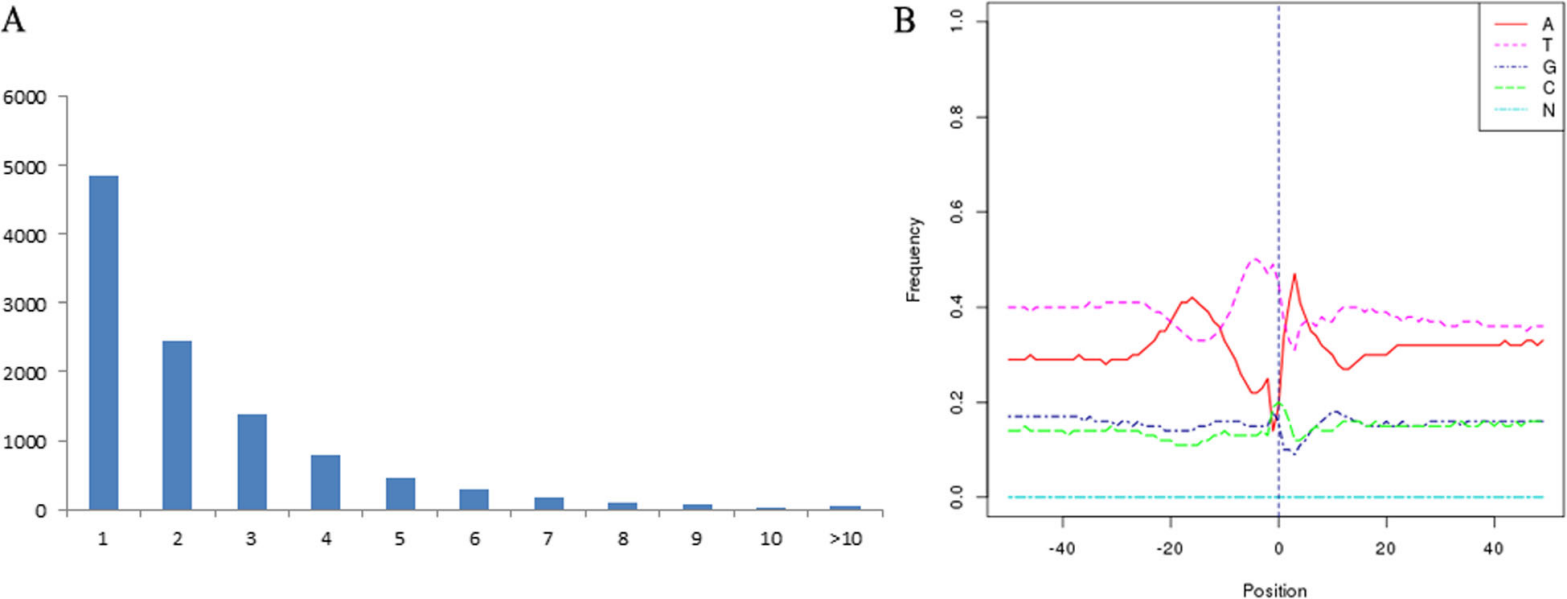
APA analysis predicted by the PacBio Sequel platform. **a** The number distribution of poly-A sites per gene. **b** Nucleotide distribution around poly-A cleavage sites

The post-transcriptional modification process of pre-mRNA to mature mRNA mainly includes the addition of a 7-methylguanosine cap at the 5′-end, intron splicing, and 3′-end formation by cleavage and polyadenylation [47]. The specific position of the poly-A tail at the 3′-end is variable, and this variation may affect the binding of microRNA or RNA-binding protein to mRNA, and the process of RNA splicing and translation. The Tapis software was used to accurately identify polyadenylation sites in anthers. By investigating the 3′-end of transcripts in our PacBio data set, 24,816 poly-A sites were detected from 10,661 genes, of which 5806 genes have alternative polyadenylation (APA) (Fig. 6b, Additional file 2: Table S12). A total of 4855 genes have at least one poly-A site, while 733 genes have more than five poly-A sites (Fig. 10a). The mean number of poly-A sites per gene was 2.33. The largest number of poly-A sites was 19, found in *BraA02g029650.3C* and *BraA04g001890.3C*. We next analyzed the nucleotide distribution of the 50 nts in upstream and downstream of all poly-A sites. Consistent with results in other species, the poly-A sites from our PacBio data set showed a nucleotide bias, with an enrichment of uracil (U) upstream and adenine (A) downstream (Fig. 10b).

## Discussion

At present, the reference genome of Chinese cabbage has been updated to version 3.0 using single-molecule sequencing. However, full-length transcripts, alterative spliced transcripts, fusion genes, and APA sites of Chinese cabbage have not been well-explored at the transcriptional level. Anthers are the male reproductive organs of plants that can produce pollen grains. The regulatory network of anther development is an extremely complex process involving a range of biological events [48]. In *Arabidopsis*, anther development has been divided into 14 stages, which make up two phases: microsporogenesis and microgametogenesis [49]. Briefly, anther development originates from stamen primordium formation, and microspore mother cells undergo meiosis to form haploid microspore tetrads. The microspores are surrounded by callose, and the release of individual microspores from the tetrads requires the action of callose enzyme secreted by the tapetum. Then, microspore wall is synthesized, followed by tapetum degradation, pollen mitosis divisions, septum cell degeneration, stomium differentiation, and finally anther dehiscence, releasing mature pollen grains. These events are relatively independent, and there is coordination in time and space. The abnormality of gene structure or expression in one of the events may cause loss of pollen function, which can generate male sterile lines. One crucial application of plant male sterility is hybrid seed production, and the advantage of hybrids is that they can increases seed yield and improve stress resistance [50, 51]. Therefore, it is necessary to investigate full-length mRNA information, providing a comprehensive view of splice isoforms in anther development.

PacBio sequencing is an effective platform for sequencing full-length transcripts because of its generation of long reads, which have an average length of 12 kb [52]. This long read length is why the PacBio sequencing platform can comprehensively analyze splice isoforms of each gene without assembly. In our work, we analyzed the full-length transcriptome of Chinese cabbage anther using the PacBio Sequel platform and yielded a total of 1,098,119 CCSs. Of these, 827,322 transcripts were identified as FLNCs, and the length of each sequencing library was consistent with the library standard (Fig. 3; Table 1). Single-molecule sequencing has a high base error rate of about 13%, mainly due to the addition of extra bases, especially in homopolymers [53]. However, as such errors occur randomly, there is no error bias, unlike that observed with NGS technology. Currently, the most common and effective method to further correct PacBio sequencing is to use high-accuracy data from an Illumina platform. With error correction using short-read RNA-Seq, 725,731 high quality FLNCs were identified to obtain 51,501 isoforms, consisting of 38,992 novel isoforms from 11,398 known genes and 3691 novel isoforms from 2682 novel genes (Additional file 2: Table S6). These results demonstrated that PacBio transcriptome sequencing can heighten the capacity to obtain full-length transcripts and enrich novel or uncharacterized isoforms or genes. Of the novel isoforms obtained, 407 high-confidence lncRNAs and 37,549 novel isoforms with predicted ORFs were identified (Additional file 2: Table S8 and Table S9). During pollen development and the fertilization process in *B. rapa*, a total of 12,501 putative lncRNAs were detected with an average length of 373 bp [42]. In our data, the mean length of predicted lncRNAs from novel isoforms was 1127 bp (Additional file 2: Table S8). Previously, the *B. rapa* genome was annotated using only ORFs, and thus there was no 5′ and 3′ UTRs defined. In 2013, Tong et al. provided a global transcriptional landscape in *B. rapa* accession Chiifu-401-42 and defined the 5′ and 3′ UTRs. The mean length of 5′ and 3′ UTRs was 139 bp and 184 bp, respectively [45]. In *Arabidopsis*, the mean length of 5′ and 3′ UTRs was 88 bp and 184 bp, respectively [54]. In our PacBio sequencing data, the mean length of 5′ and 3′ UTRs from novel isoforms with predicted ORF was 788 bp and 641 bp, respectively (Fig. 8c, d).

In addition to capturing full-length transcripts, another advantage of PacBio sequencing is the ability to detect AS events, which play a crucial role in regulating cellular molecules, cellular physiology, and developmental pathways [45, 55, 56]. The proportion of AS genes in rice, maize, *B. rapa*, and *A. thaliana* is 33, 37, 42, and 61%, respectively [57–59]. Limited by short reads, previous studies of the transcriptome using NGS technology have only been able provide individual splice junctions, while PacBio sequencing technology can be applied to alternatively spliced forms for each mRNA [39]. IR is the most common event in various genomes, which supports an intron-definition mechanism for pre-mRNA splicing [60]. In our study, we collected anthers from all developmental stages to harvest relatively comprehensive spliced isoforms. However, we detected a total of 453,270 AS events, and the majority of AS events were XSKIP (Fig. 9a). Previous studies indicated that alternative spliced transcripts have tissue-specific expression in various plants [61–64]. For novel splice junctions in *B. rapa*, 34.4% of alternative spiced transcripts were detected in only one tissue [45]. Therefore, differences in the prevalence of AS events may be related to tissue specificity. Those findings illustrate the complexity of the anther-specific transcriptome. Unfortunately, the expression levels of transcripts detected by PacBio sequencing have not been analyzed, and there is no way to analyze the expression pattern of different isoforms from one gene caused by AS events.

Taking the model plant *Arabidopsis* as an example, the key regulatory genes during anther development have been quite extensively reported, and are mainly involved in microsporogenesis, tapetum layer formation, callose layer development, pollen wall formation, and anther dehiscence [65]. Chinese cabbage and *Arabidopsis* both belong to the Brassicaceae family, and so are closely related and have high sequence similarity. Therefore, we complied 34 genes that have been confirmed to be involved in anther development in *Arabidopsis* (Additional file 2: Table S13). In addition to the three whole genome duplications (WGDs) that occurred in Brassicaceae, the Brassica genome has undergone an additional ancient triplication, accompanied by gene fractionation [38]. Thus, based on the best BLASTX search in the Brassica database, we obtained 53 annotated genes from the PacBio annotation data (Additional file 2: Table S13). Of these genes, *AGAMOUS* (*AG*), *SPOROCYTELESS/NOZZLE* (*SPL*/*NZZ*), *BARELY ANY MERISTEM1/2* (*BAM1*/*2*), *Extra sporogenous cells*/*Excess microsporocytes 1* (*EMS1/EXS*), *SOMATIC EMBRYOGENESIS RECEPTOR-LIKE KINASE 1* (*SERK1*), and *TAPETUM DETERMINANT 1* (*TPD1*) were annotated for microsporogenesis in the early stages of anther development. For tapetal development and programmed cell death (PCD), the key genes detected were *Arabidopsis thaliana MYB DOMAIN PROTEIN 80/103* (*AtMYB80*/*AtMYB103*), *Dysfunctional Tapetum 1* (*DYT1*), *Tapetal Development and Functional 1* (*TDF1*), *Aborted microspores* (*AMS*), and *Male sterility* (*MS1*). For pollen exine formation, *Callose synthase 5* (*CALS5*), *Cyclin-dependent kinase G1* **(***CDKG1*), *AUXIN RESPONSE FACTOR17* (*ARF17*), *No exine formation 1* (*NEF1*), *Ruptured pollen grains 1* (*RPG1*), *Defective in exine formation 1* (*DEX1*), *No primexine and plasma membrane undulation* (*NPU*), *CYP703A2, Acetyl-coenzyme A synthetase 5* (*ACOS5*), *MALE STERILITY 2* (*MS2*), *Less adherent pollen5* (*LAP5*), and *ATP-binding cassette G26* (*ABCG26/WBC27*) were detected. For pollen intine formation, *Cellulose synthase 1/3* (*CESA1*/*3*), *ARABINOGALACTAN PROTEIN 6/11* (*APG6*/*11*), and *Fasciclin-like arabinogalactan protein 3* (*FLA3*) were identified. For anther dehiscence, *MYB DOMAIN PROTEIN 26* (*MYB26*), and *NAC SECONDARY WALL THICKENING PROMOTING FACTOR 1* (*NST1*) were generated. Moreover, some of the loci were found to contain different alternatively spliced isoforms in our PacBio data set. For example, two loci (*BraA07g036270.3C* and *BraA07g029410.3C*) were annotated as *SERK1,* which is important for anther cell specification, but only *BraA07g036270.3C* expressed two alternatively spliced isoforms. As early as the meiosis phase, the callose layer begins to deposit outside the plasma membrane of microspore mother cells, which is the initiation of pollen wall development. In *Arabidopsis*, 12 *CALS* genes were identified, of which *CALS5* plays an important role in callose synthesis during the tetrad period. In mutant *cals5*, callose is insufficiently produced around microspores, resulting in defects in primexine formation and subsequently affecting the deposition of sporopollen in the pollen exine [66]. In our data, *CALS5* (*BraA09g010050.3C*) had ~ 1065 spliced variants, and XMSKIP predominated in AS models. For primexine formation, two loci (*BraA10g025410.3C* and *BraA02g004840.3C*) were annotated as *NEF1*. Two AS events, IR and XAE, were detected in *BraA10g025410.3C*, and XSKIP was found in *BraA02g004840.3C*. In *Arabidopsis*, multiple *CESA* genes encoding a cellulose synthase associated with pollen intine formation were cloned; the knockout mutant of *cesa1* and *cesa3* exhibited the gametophytic sterility phenotype, with abnormal pollen wall [67]. Both the annotated *CESA1* and *CESA3* in Chinese cabbage contained two loci each. *BraA01g005650.3C* was one of the *CESA1* loci, of which 40 alternatively spliced isoforms were detected, including twelve IR, six XIR, twelve XMSKIP, and ten XAE. *CESA1* (*BraA03g057280.3C)* had twelve alternatively spliced isoforms, consisting eight IR, two XIR, and two XAE. Similarly, two loci were annotated as *CESA3* (*BraA03g002020.3C* and *BraA02g001600.3C*). Eight AS events: six IR and two XAE were detected in *BraA03g002020.3C*, and two IR were detected in *BraA02g001600.3C*. Our research identified AS events in key genes active during anther development in Chinese cabbage.

## Conclusions

Full-length transcriptome technology was used to explore the transcripts and splice isoforms present during anther development in Chinese cabbage. A total of 51,501 isoforms were identified using the PacBio Sequel platform. Meanwhile, 453,270 AS events were detected, and XSKIP events were found to have occurred extensively in anther. A total of 53 key genes active during anther development were detected in our PacBio sequencing, of which eight annotated loci had alternatively spliced isoforms. Additionally, 104 fusion transcripts and 24,816 poly-A sites were also predicted in this study. These new findings provide a valuable resource for complete characterization of anther-specific transcriptome data and improved Chinese cabbage genome annotation.

## Methods

### Plant material

The excellently Chinese cabbage DH line ‘FT’ was independently created by our laboratory (Liaoning Key Lab of Genetics and Breeding for Cruciferous Vegetable Crops) using isolated microspore culture technology. The DH line ‘FT’ is characterized by extremely early maturity, heat resistance, ovoid leaf head, and white petals (Fig. 1a). In August 2018, the DH line ‘FT’ seeds were placed in a 4 °C refrigerator for vernalization, and then sown in a greenhouse at Shenyang Agricultural University. At the full-bloom stage, three plants with consistent growth were randomly selected, and the whole buds of a complete inflorescence from each plant were individually collected in pieces of aluminum foil (Fig. 1b). Then, the anthers from each bud were detached, frozen in liquid nitrogen, and stored at − 80 °C prior to SMRT sequencing (Fig. 1c).

### PacBio library construction and sequencing

Total RNA from the three samples was extracted using Trizol reagent (Invitrogen, CA, USA). RNA purity and integrity was monitored by NanoPhotometer® spectrophotometer (IMPLEN, CA, USA) and a Bioanalyzer 2100 system (Agilent Technologies, CA, USA). RNA contamination was assessed by 1% agarose gel. RNA concentration was detected using a Qubit® 2.0 Fluorometer (Life Technologies, CA, USA). Equimolar rations of the total RNA from each sample were mixed together. The full-length cDNA was prepared using a SMARTer™ PCR cDNA Synthesis Kit (Takara Biotechnology, Dalian, China). Size fractionation (1–2, 2–3, and > 3) of full-length cDNA was achieved using the BluePippin™ Size Selection System (Sage Science, Beverly, MA). The filtered full-length cDNAs were subjected to re-amplification, end repair, SMRT adapter ligation, and exonuclease digestion. After secondary screening by BluePippin™, three SMRTbell libraries were constructed with the Pacific Biosicences DNA Template Prep Kit 2.0. Library quantification and size was measured using a Qubit® 2.0 Fluorometer (Life Technologies, CA, USA) and Bioanalyzer 2100 system (Agilent Technologies, CA, USA). Subsequently, SMRT sequencing was performed on a PacBio Sequel platform by Frasergen Bioinformatics Co., Ltd. (Wuhan, China).

### Illumina RNA-Seq library construction and sequencing

In parallel, the quantity and purity of equally mixed RNA were analyzed using Bioanalyzer 2100 and RNA 6000 Nano LabChio Kit (Agilent, CA, USA). Poly (A) mRNA was isolated by poly-T oligoattached magnetic beads (Invitrogen). Following fragmentation, the cleaved RNA fragments were reverse-transcribed into a cDNA library following treatment with the mRNASeqample Preparation Kit (Illumina, San Diego, USA). After assessing the library quality, we performed PE300 sequencing on an Illumina Hiseq 2500 at the LC Sceiences (Hangzhou, China) following the vendor’s recommended protocol.

### Quality filtering and error correction

PacBio raw reads were preprocessed and filtered using SMRT Link v5.0. Briefly, CCSs were generated from the subread SAM file with the following parameters: minimum subread length = 50; minimum number of passes = 1, minimum predicted accuracy = 0.8, and minimal read score = 0.65. Then, CCSs were classified into either full length or non-full length reads, by assessing the presence of the 5′ and 3′ adapters and poly (A) tail. FLNC reads were full length CCSs containing all three elements, with no additional copies of the adapter sequence within the DNA fragment.

The high-quality Illumina short reads were used to error correction for FLNC reads. The proovread software v2.12 was widely and efficiently applied for correcting FLNC sequences by iterative short read consensus [68]. Using GMAP^2^ software, the FLNC sequences before and after error correction were compared to the *B. rapa* v3.0 reference genome [69, 70] with “—no-chimeras and –n 100” to calculate the PID values, including global PID and local PID (Fig. 2b). The higher the PID values, the more consistent the sequencing data was with the reference genome. The PID values of the genomic comparison before and after error correction were separately counted, and the higher PID values were updated. Then, the uniquely mapped FLNC sequences with high PID (global PID > 95% and local PID > 97%) were used to annotate loci and isoforms.

### Gene loci and isoform finding

Gene loci and isoforms were identified based on the alignment position of the corrected FLNC reads. For loci, two transcripts that overlapped by at least 20% of their initiation sites on the same strand, and had at least one exon overlap of more than 20%, were considered to be the same loci transcript. These same loci transcripts were further analyzed for isoform identification. The process mainly included the removal of redundant transcripts and the filtration of low-reliability transcripts. The redundant transcripts were removed as follows: firstly, if all the splicing sites of the same loci transcripts were identical, they could be considered one isoform; secondly, if one isoform was degraded at the 5′ terminal region, but the remaining region was consistent with other isoforms, it should be filtered out. For false positives, when the global PID < 99%, each isoform structural model must supported with least two FLNC reads; otherwise, if there was only one sequence, then all junction sits of the sequence were fully supported by the genomic annotation or Illumina RNA-Seq data.

### Novel gene and isoform identification

The above gene loci and isoforms were compared with the reference annotation to identify known genes and isoforms, as well as novel genes and isoforms. A sequenced gene was determined to be a novel gene by satisfying any of the following criteria: (i) There is no overlap or there is an overlap of less than 20% of the annotated genes; or (ii) the overlap with the annotated gene is greater than 20%, but the gene direction is inconsistent. In addition, if the sequenced isoform contained one or more new splicing sites, or if the sequenced isoform and annotated isoform were not both single-exon, it was considered to be a novel isoform.

### Functional annotation

The novel isoforms were annotated by NR, KOG, KO, and Swiss-Prot databases with Diamond [71, 72]. KEGG pathways were searched by KOBAS v2.0 [73]. GO annotations were performed by BLASTX v2.2.26 and BLAST2GO v2.3.5 software [74].

### LncRNA and ORF identification

To identify LncRNA, novel isoforms from known genes or novel isoforms from novel genes obtained by PacBio data were first searched against NR, KOG, KO, and Swiss-Prot databases with default parameters. The isoforms that had BLAST hits with 1E-5 were filtered out, and the remaining isoforms were further evaluated for protein-coding capacity by CPAT v1.2.2 (http://lilab.research.bcm.edu/cpat/).

To predict ORFs, transDecoder software was used to identify potential coding sequences (http://transdecoder.sf.net). By default, the length of ORFs predicted by TransDecoder.LongOrfs was at least 100 amino acids. To improve the sensitivity of ORFs, possible ORF-translated proteins were aligned to the Swiss-Prot database by BlastP for homologous protein identification. Simultaneously, protein domain identification was determined from the Pfam database by Hmmscan [75, 76]. Subsequently, TransDecoder. Predict was used to filter all predicted ORFs based on the above results, and retained ORFs that have homology to the Swiss-Prot database or with the same domain.

### AS, fusion transcript, and APA identification

Alternative splicing (AS) events were ascertained using ASprofile software [77]. The splice types, (M) SKIP, (M) IR, AE, X (M) KIP, X (M) IR, and XAE were classified and characterized by comparing different isoforms at the same gene loci using ASprofile (Fig. 2c). Fusion transcripts were those where the 5′ and 3′ sequences mapped to two or more gene loci in the reference genome, corresponding to the 5′ partner and 3′ partner genes. The iso-seq fusion transcripts detection software, self-developed by Frasergen Inc. (Wuhan, China), was used for fusion gene detection. A schematic diagram of the software is shown in Fig. 2d. Poly-A site was an important post-transcriptional modification of RNA. The reliable APA sites were obtained by Tapis software [33].

### RT-PCR validation

Total RNA from floral organs of the DH line ‘FT’, including anthers, sepal, filament, petal, and pistil were extracted and mixed as described above. Reverse transcription was conducted using the FastQuant RT Super Mix (TIANGEN, China). RT-PCR was performed in 10 μl volumes containing 50 ng DNA, 1.0 μl of 10 Taq Reaction Buffer (containing Mg2^+^), 0.8 μl of 2.5 mM dNTP, 1 μl each of 0.5 μm forward and reverse primers, and 1 U of Taq DNA polymerase (TIANGEN, China). The amplification was performed on an iCycler thermocycler (Bio-Rad, USA) with the following cycling parameters: initial denaturation at 95 °C for 5 min, and 35 cycles of 95 °C for 30 s, 56 °C for 30 s, and 72 °C for 30 s, with a final extension at 72 °C for 10 min. Gene-specific primers were designed with Primer Premier 5.0 by GENEWIZ (Suzhou, China). PCR products were analyzed on 2% agarose gels and followed by Sanger sequencing. All primers are listed in Additional file 2: Table S14.

## Supplementary information

**Additional file 1 Figure S1.** RT-PCR validation of AS events (1–3) and fusion transcripts (4–6). M, DNA Marker DL2000; A, anther; S, sepal; F, filament; Pe, petal; Pi, pistil; 1, m54191_180531_084316/71238183/3459_97_CCS; 2, m54191_180531_084316/15467311/43_3034_CCS; 3, m54045_180508_172253/21365668/2097_84_CCS; 4, *BraA03g009520.3C*; 5, *BraA01g012300.3C*; 6, *BraA02g020980.3C*.

**Additional file 2 Table S1.** Summary of polymerase reads from the PacBio Sequel platform. **Table S2.** Summary of subreads from the PacBio Sequel platform. **Table S3.** Summary of CCSs from the PacBio Sequel platform. **Table S4.** Global PID statistics before and after sequencing error correction. **Table S5.** Evaluation of full-length transcripts in the PacBio data set. **Table S6.** Classification of loci and isoforms mapped to the reference genome. **Table S7.** Functional annotation of all novel isoforms by the PacBio Sequel platform. **Table S8.** Information of lncRNAs from the PacBio Sequel platform. **Table S9.** ORF information detected by the PacBio Sequel platform. **Table S10.** Splice isoforms detected by PacBio Sequel platform. **Table S11.** Fusion genes detected by the PacBio Sequel platform. **Table S12.** Poly-A sites detected by the PacBio Sequel platform. **Table S13**. Anther and pollen development related genes in *B. rapa* genome v3.0. **Table S14**. Primers used for RT-PCR validation.

## Data Availability

The datasets supporting the conclusions of this article are included within the article and its additional files. We deposited the raw SMRT data in the Sequence Read Archives (SRA) of the National Center for Biotechnology Information (NCBI) under the accession numbers SRR10259626, SRR10259627 and SRR10259628 of Bioproject PRJNA576779. The Illumina RNA-Seq data was uploaded to the SRA under the accession number SRR10247439 of the Bioproject ID PRJNA576332. Genomic sequences and gene annotation information of *B.rapa* are downloaded online at http://brassicadb.org/brad/datasets/pub/Genomes/Brassica_rapa/V3.0/.

## Abbreviations

APA: alternative polyadenylation
AS: alternative splicing
CCS: circular consensus sequence
FLNC: full-length non-chimeric
LncRNA: long non-coding RNA
NGS: next-generation sequencing
ORF: open reading frame
PID: percentage-of-identity
SMRT: single-molecule real-time sequencing
