# Supplementary figures and images for: Single-molecule real-time sequencing facilitates the analysis of transcripts and splice isoforms of anthers in Chinese cabbage (Brassica rapa L. ssp. pekinensis)
Source: BMC Plant Biol. 2019 Nov 27;19:517. doi: 10.1186/s12870-019-2133-z (PMC6880451; doi:10.1186/s12870-019-2133-z)

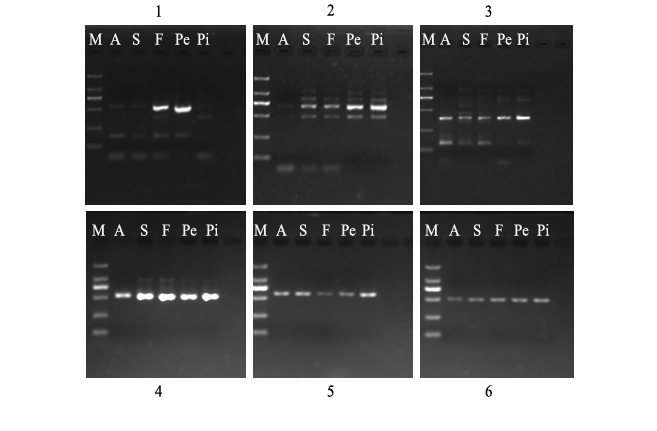

Supplement: Supplementary file 1 — Additional file 1 Figure S1. RT-PCR validation of AS events (1–3) and fusion transcripts (4–6). M, DNA Marker DL2000; A, anther; S, sepal; F, filament; Pe, petal; Pi, pistil; 1, m54191_180531_084316/71238183/3459_97_CCS; 2, m54191_180531_084316/15467311/43_3034_CCS; 3, m54045_180508_172253/21365668/2097_84_CCS; 4, BraA03g009520.3C; 5, BraA01g012300.3C; 6, BraA02g020980.3C. [file 12870_2019_2133_MOESM1_ESM.tif]
